# Enhancement of Physicochemical and Functional Properties of Chicken Breast Protein Through Polyphenol Conjugation: A Novel Ingredient for Protein Supplements

**DOI:** 10.3390/molecules30030448

**Published:** 2025-01-21

**Authors:** Woo-Young Son, Jun Hwang, Ju-Hyo Park, Ji-Han Kim, Raise Ahmad, Kyeong-Soo Kim, Hyun-Wook Kim

**Affiliations:** 1 Division of Animal Bioscience & Integrated Biotechnology, Gyeongsang National University, Jinju 52828, Republic of Korea; sonwy001223@naver.com (W.-Y.S.); hwangjun1116@naver.com (J.H.); 2 Jungdam Co., Ltd., Suwon 16602, Republic of Korea; jungdam1004@naver.com; 3 Smart Foods, Ag Research, Palmerston North 4410, New Zealand; jihan.kim@agresearch.co.nz (J.-H.K.); raise.ahmad@agresearch.co.nz (R.A.); 4 Department of Pharmaceutical Engineering, Gyeongsang National University, Jinju 52725, Republic of Korea; soyoyu79@gnu.ac.kr; 5 Department of GreenBio Science, Gyeongsang National University, Jinju 52725, Republic of Korea

**Keywords:** antioxidant capacity, emulsifying capacity, chicken breast protein, protein digestibility, protein–polyphenol conjugate characterization

## Abstract

Polyphenol conjugation has emerged as a promising approach to enhance the technological properties and physiological benefits of food proteins. This study investigated the effects of polyphenol conjugation on the technological properties, antioxidant capacity, and in vitro digestibility of chicken breast (CB) proteins. Conjugation with (-)-epigallocatechin 3-gallate (EGCG) and tannic acid (TA) significantly reduced sulfhydryl content. EGCG conjugates exhibited higher turbidity and greater molecular weight aggregates (>245 kDa). Fourier-transform infrared spectroscopy (FTIR) revealed alterations in protein secondary structures, with shifts in amide I and II bands. Polyphenol conjugation significantly enhanced the water-holding capacity of chicken muscle proteins, particularly for CB-TA (3.29 g/g) and CB-EGCG (3.13 g/g) compared to the control (2.25 g/g). The emulsion stability index improved notably in CB-EGCG (96.23 min) and CB-TA (87.24 min) compared to the control (69.05 min). Color analysis revealed darker and more intense hues for CB-EGCG, while CB-TA maintained a lighter appearance, making it potentially preferable for industrial applications requiring neutral-colored powders. Moreover, polyphenol conjugation could enhance antioxidant capacity, particularly in conjugates with EGCG (*p* < 0.05). In vitro protein digestibility remained comparable across treatments (*p* > 0.05). Our findings could indicate the potential of chicken muscle protein–polyphenol conjugates as innovative ingredients for high-quality protein supplements.

## 1. Introduction

Protein supplements, once regarded as niche products for professional athletes, are now widely recognized as regular dietary tools for increasing protein intake among general consumers seeking muscle growth, dietary supplementation, and weight management [1]. In developed countries experiencing rapid population aging, the demand for senior-friendly protein supplements has grown significantly as older adults aim to meet their protein requirements to maintain health and prevent sarcopenia [2]. The global protein supplement market was valued at USD 26.1 billion in 2023 and is projected to grow at a compound annual growth rate (CAGR) of 8.81% through 2032 [3]. This consumer trend is driven by the challenge of consuming the recommended dietary allowance of protein through conventional diets and the desire for higher protein intake to promote muscle building. Nevertheless, prolonged excessive protein consumption has been associated with adverse effects on physical health, such as impaired kidney function [1]. As a result, modern consumers prefer to consume high-quality proteins rich in essential amino acids, offering excellent bioavailability to meet specific dietary needs. Despite the prevalence of soy and dairy proteins in most commercial supplements, a significant knowledge gap remains regarding meat-derived protein supplements, which could provide highly digestible proteins with balanced amino acid profiles.

Increased personal income levels and population growth are the main factors driving the rise in global meat consumption, and the patterns of meat consumption (production methods, preferred animal species, and ways of consumption) are becoming increasingly diverse due to social and environmental factors [4]. One of the critical features of these changing meat consumption patterns is the continued increase in poultry consumption for several reasons, including its low cost, health benefits, and versatility in cooking [5]. Specifically, poultry meat, such as chicken breast, is recognized as an ideal resource for developing senior-friendly meat products due to its nutritional benefits (high in protein and low in saturated fat), physiological advantages (high digestibility), and sensory appeal (tender texture) [6,7]. Furthermore, in the case of South Korea, this growing demand has spurred the development of dried chicken breast powder as a protein supplement, catering to consumers seeking convenience in storage and consumption. In this regard, chicken breast powder, due to its processed nature, may exhibit differences in protein digestibility and bioavailability compared to unprocessed chicken, potentially due to structural modifications during processing.

Humans commonly consume fruits and vegetables rich in polyphenols and protein-rich foods such as meat in their daily diets, and this may occur more frequently when health is a priority. Polyphenol intake is well known to provide numerous physiological benefits, including antioxidant, anti-inflammatory, and antimicrobial effects, cardiovascular protection, cancer prevention, blood sugar regulation, neuroprotection, gut health promotion, weight management support, and enhanced skin health [8,9,10]. However, many previous studies have found that polyphenols can bind to food proteins or endogenous digestive enzymes in the body, reducing the digestibility of consumed proteins [9,10]. However, recent research has indicated the formation of protein–polyphenol conjugates to improve the bioavailability of these two important components and enhance their processing and functional properties, especially antioxidant capacity [11]. Previously, it has been well-documented that polyphenols can bind to proteins through covalent and non-covalent interactions, such as hydrogen bonding and hydrophobic interactions. In particular, conjugation under alkaline conditions (around pH 9.0), one of the representative non-enzymatic methods, can effectively form covalent bonds between proteins and polyphenols by generating semiquinone radicals from polyphenols [10]. Moreover, conjugating muscle proteins, primarily myofibrillar proteins, with polyphenols can noticeably improve their processing properties (e.g., solubility, emulsifying capacity, and gel-forming ability) and antioxidant capacity [11,12,13].

Taken together, it could be hypothesized that polyphenol conjugation with chicken breast muscle proteins can enhance processing characteristics, antioxidant capacity, and bioaccessibility. Recently, Xu et al. [12] reported that the conjugation of glycated chicken myofibrillar protein with polyphenols ((-)-epigallocatechin-3-gallate (EGCG), catechin, or gallic acid), via covalent bond formation, improved their antioxidant capacity and thermal stability. Moreover, Xu et al. [13] suggested that ternary conjugates of chicken myofibrillar proteins, dextran, and polyphenols exhibited enhanced emulsifying capacity and oxidation stability. While previous studies have aptly demonstrated the benefits of polyphenol conjugation in improving the processing characteristics and antioxidant capacity of chicken breast-derived proteins, information on bioaccessibility, a critical factor for the application of these conjugates as novel ingredients in protein supplements, remains limited. Thus, it is necessary to understand bioaccessibility, which refers to the extent to which protein–polyphenol conjugates become available for absorption during digestion.

From an industrial perspective, the diversity of muscle proteins and their varying solubilities present obvious technical and cost challenges in isolating and purifying individual proteins. Despite advances in meat processing technologies, the development of powdered meat products for specific protein supplementation has been limited. Recent advancements in drying techniques, such as freeze-drying and microwave vacuum drying, have enabled the production of powdered meat without the significant thermal denaturation of muscle proteins. Moreover, microwave vacuum drying is a faster and more energy-efficient method, suitable for scaling industrial processes, though it may lead to moderate nutrient loss due to localized heating.

Therefore, this study aims to evaluate the effects of polyphenol conjugation (EGCG and tannic acid) on the processing characteristics, antioxidant capacity, and in vitro digestibility of chicken breast powder to determine its potential as a novel ingredient for protein supplements.

## 2. Results and Discussion

### 2.1. Confirmation and Characterization of Protein–Polyphenol Conjugate

#### 2.1.1. Sulfhydryl Content and Turbidity

**Figure 1:**
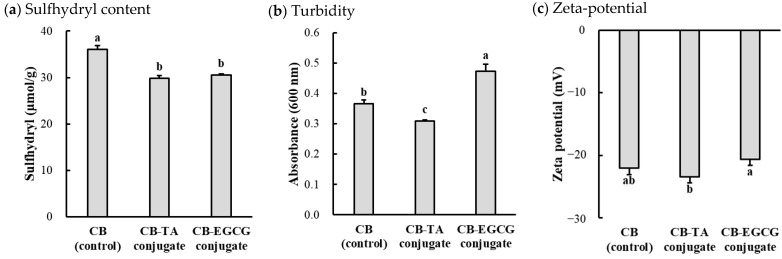
Sulfhydryl content (**a**), turbidity (**b**), and zeta-potential (**c**) of chicken muscle protein–polyphenol conjugates. CB, chicken breast powder (as control); CB-TA, chicken breast protein–tannic acid conjugate; CB-EGCG, chicken breast protein–EGCG conjugate. a–c Means with the same letters between treatments are not significantly different (*p* > 0.05).

The formation intensity of protein-polyphenol conjugates was confirmed by sulfhydryl content (Figure 1a) and spectrophotometric turbidity (Figure 1b). The conjugation with tannic acid or EGCG significantly reduced the sulfhydryl content of chicken muscle proteins. No significant difference in the sulfhydryl content was found between the conjugate treatments (CB-TA vs. CB-EGCG). Sulfhydryl in amino acids, namely the free thiol groups (-SH) of proteins, can form covalent bonds with polyphenols. Thus, the decreased sulfhydryl content in this study could be evidenced by increased protein–polyphenol conjugates. Turbidity is commonly used as an indicator of the protein–polyphenol conjugate formation based on spectrometric changes. In this study, the type of polyphenols significantly affected the turbidity of conjugated chicken muscle proteins; CB-EGCG showed higher turbidity compared to CB control, but the turbidity of CB-TA was slightly lower than that of the control (*p* < 0.05). These results were likely that conjugation with EGCG can induce protein aggregation due to their tendency to crosslink protein molecules [14]. However, the CB-TA conjugate exhibited the lowest turbidity (*p* < 0.05), which might be explained by solubility improvement. Due to its hydrophilic properties, tannic acid may form smaller, more soluble conjugate complexes, preventing excessive aggregation [15]. More comprehensively, it will be possible to identify the fundamental reasons for turbidity changes by comparing differences in the structural identification of conjugates depending on the polyphenols.

#### 2.1.2. Zeta-Potential

Zeta potential, an important measure of particle surface charge, reflects the stability of colloidal systems by indicating the degree of electrostatic repulsion between particles. Particles with absolute zeta potential values greater than ±30 mV are considered highly stable dispersions due to their strong repulsion, while values below ±5 mV suggest weak repulsion and higher cohesiveness [16,17]. This parameter is widely used to predict the stability and cohesive behavior of colloidal solutions in food and biopolymer systems [18]. In this study (Figure 1c), CB control exhibited a zeta potential of −22.0 mV, showing sufficient repulsion for stable dispersion. Compared to the control, CB-TA exhibited a lower zeta potential of −23.4 mV (*p* < 0.05), suggesting an increase in surface negative due to the conjugation of phenolic groups in tannic acid. This is consistent with the findings that polyphenols enhance surface charge through covalent bonding, resulting in increased dispersion stability [19]. Conversely, CB-EGCG exhibits a zeta potential of −20.6 mV, indicating relatively weaker electrostatic repulsion compared to the control. The smaller size of the EGCG and its selective interaction with specific amino acid residues can reduce the surface charge density while inducing stereoscopic effects [20]. This can distinguish CB-EGCG from more stable CB-TA in favor of coagulation under certain conditions. Moreover, the results for the zeta potential could be correlated closely with the turbidity results (Figure 1b). More specifically, CB-EGCG, with the highest turbidity, also had the lowest negative zeta potential, indicating a weak electrostatic repulsion that allows particles to aggregate more easily. This relationship denotes the important role of zeta potential in controlling the clustering behavior, as the surface charge reduction reduces the electrostatic force required to maintain dispersion stability [18]. On the other hand, CB-TA, with the highest negative zeta potential, showed the lowest turbidity, suggesting an improved dispersion due to stronger repulsive forces between particles.

#### 2.1.3. Protein SDS-PAGE

**Figure 2:**
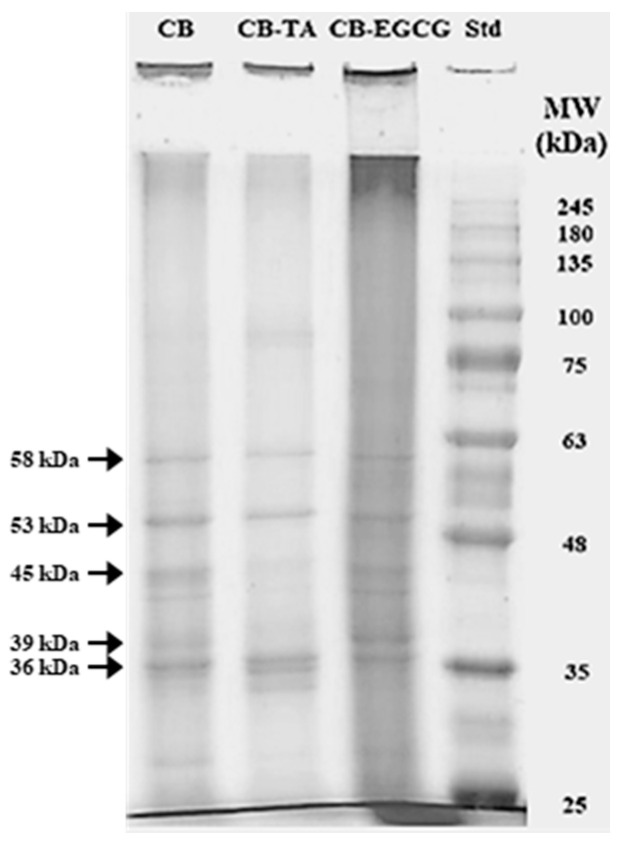
A representative photo of protein SDS-PAGE (5% stacking gel and 8% separating gel) of chicken breast protein–polyphenol conjugates. CB, chicken breast powder (as control); CB-TA, chicken muscle protein–tannic acid conjugate; CB-EGCG, chicken muscle protein–EGCG conjugate; Std, protein standard marker. One hundred micrograms of protein were loaded in each sample.

Protein SDS-PAGE was performed to observe the effects of polyphenol conjugation on the molecular weight distribution of chicken breast proteins (Figure 2). Distinct patterns of protein bands were found. In CB control, several prominent bands were observed, with the major bands appearing at approximately 36 kDa, 39 kDa, 45 kDa, 53 kDa, and 58 kDa, possibly corresponding to glyceraldehyde phosphate dehydrogenase (36 kDa), aldolase (39 kDa), actin (45 kDa), desmin (53 kDa), and phosphoglucose isomerase (58 kDa), respectively [21]. In the CB-TA conjugate, the intensity of the 36 kDa, 45 kDa, and 53 kDa protein bands was noticeably reduced compared to the control. This reduction in band intensity suggests that these proteins might be polymerized, increasing band intensity around 90 kDa, indicating the formation of higher molecular weight complexes. This exhibits that tannic acid conjugation led to the formation of protein–polyphenol aggregates. In the CB-EGCG conjugate, an overall decrease in band intensity was observed, particularly excluding the formation of high-molecular-weight aggregates above 245 kDa. This denotes that EGCG conjugation led to extensive protein aggregation. Despite tannic acid having a larger molecular weight than EGCG, EGCG exhibited greater reactivity with proteins, forming multiple molecular bonds with a single protein and inducing significant aggregation [22]. Our finding on the protein SDS-PAGE indicates that both tannic acid and EGCG significantly modify the molecular weight distribution of chicken muscle proteins; moreover, conjugation with EGCG may induce stronger crosslinking and aggregation effects on chicken muscle proteins.

#### 2.1.4. FTIR

**Figure 3:**
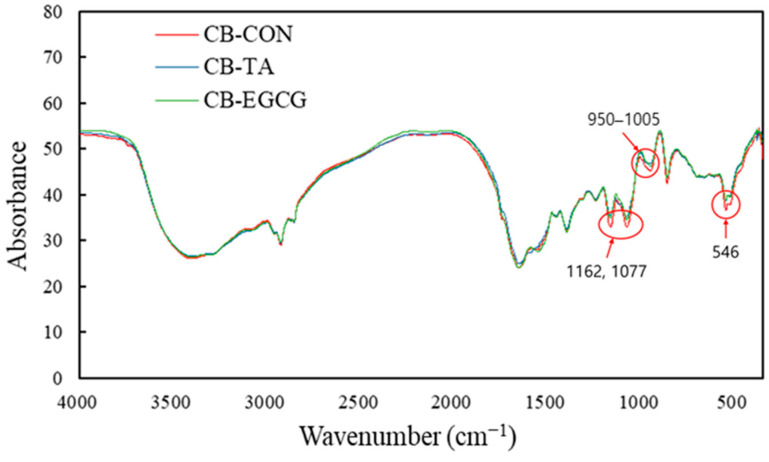
Infrared absorption spectra obtained from chicken breast powder (CB, as control), chicken muscle protein–tannic acid conjugate (CB-TA), and chicken muscle protein–EGCG conjugate (CB-EGCG).

The FTIR spectra of chicken muscle protein–polyphenol conjugates are shown in Figure 3. CB-TA and CB-EGCG conjugates exhibit reduced absorption peaks compared to CB control in the regions around 1162 and 1077 cm^−1^, as well as between 950 and 1005 cm^−1^. These reductions indicate effective binding interactions between polyphenols and the protein matrix. The region from 1200 to 900 cm^−1^ corresponds to decreases in C-O and C-O-C bonds or O-H vibrations, notifying the weakening or rearrangement of existing bonds as a result of protein–polyphenol interactions [23]. A broad absorption band around 3270 cm^−1^, attributed to O-H stretching vibrations, is observed in all samples. The presence of this band indicates extensive hydrogen bonding and the incorporation of polyphenols can enhance these interactions, potentially improving the hydration and thermal stability of the protein matrix. The amide I and amide II bands of the conjugates appear at approximately 1655 cm^−1^ and 1550 cm^−1^, respectively, implying that EGCG induces changes in protein secondary structures through vibrational stretching of protein bonds. These bands correspond to specific frequencies associated with α-helices and β-sheets, overlapping signals reflecting secondary structural changes in polypeptide chains [24]. Such structural modifications are further supported by frequency shifts attributed to secondary structure alterations in polypeptides [25].

### 2.2. Technological Properties

**Figure 4:**
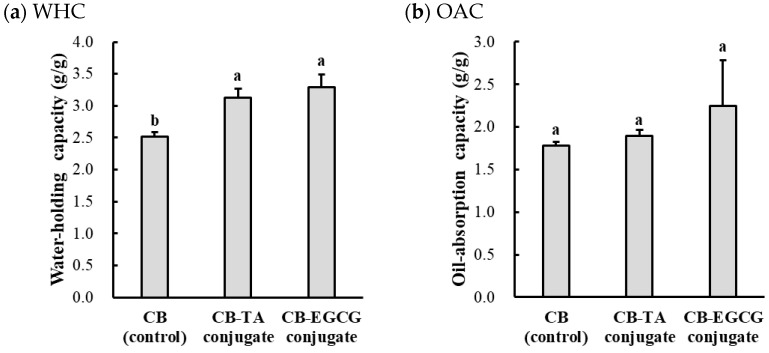

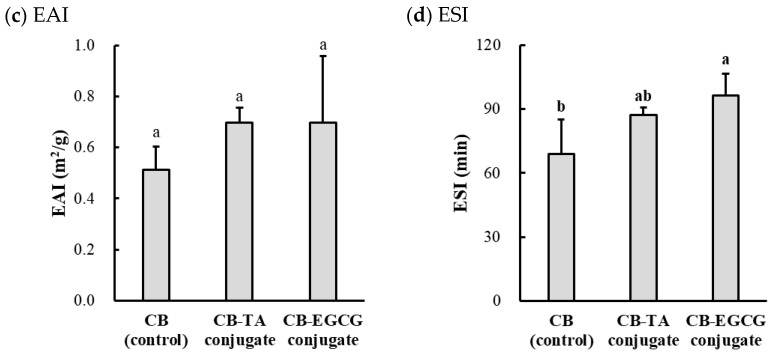
Water-holding capacity (WHC, (**a**)), oil absorption capacity (OAC, (**b**)), emulsion activity index (EAI, (**c**)), and emulsion stability index (ESI, (**d**)) of chicken breast muscle protein–polyphenol conjugates. CB, chicken breast powder (as control); CB-TA, chicken muscle protein–tannic acid conjugate; CB-EGCG, chicken breast–EGCG conjugate. a–b Means with the same letters between treatments are not significantly different (*p* > 0.05).

The technological properties of chicken muscle protein–polyphenol conjugates, such as the water-holding capacity (WHC), oil absorption capacity (OAC), emulsion activity index (EAI), and emulsion stability index (ESI), are shown in Figure 4.

#### 2.2.1. WHC and OAC

When compared to the WHC of the CB control (2.25 g/g), CB-TA and CB-EGCG showed significantly increased WHC of 3.29 g/g and 3.13 g/g, respectively (*p* < 0.05). The WHC, which reflects the amount of water that protein–polyphenol conjugates can retain without releasing liquid, was significantly higher for CB-TA and CB-EGCG at 3.29 (g/g) and 3.13 (g/g), respectively, compared to the CB control at 2.52 (g/g) (*p* < 0.05). The increased WHC of the conjugates suggests that polyphenol binding could enhance the hydrophilic behavior of the protein matrix, which may be due to the hydroxyl groups promoting hydrogen bonding with water molecules [26]. These results are consistent with previous findings denoting that polyphenols alter the surface characteristics of proteins, resulting in enhanced water-holding capacity and flexibility [27,28]. Regarding OAC, a numerically increasing trend was observed for chicken muscle protein–polyphenol conjugates, but there was no significant difference.

#### 2.2.2. Emulsifying Capacity (EAI and ESI)

The EAI, an indicator of the initial emulsification ability of the raw material, showed a slight increase for CB-TA and CB-EGCG conjugates, while there was no significant difference. These results indicate that polyphenol conjugation improves interfacial adsorption, but the extent of improvement may vary depending on the structural modifications induced by specific polyphenols. The larger molecular size and multiple tannic acid binding sites could lead to a broader surface coverage. In contrast, the smaller size and selective binding of EGCG may induce localized effects that improve protein flexibility and interfacial activity [29]. ESI, which evaluates the stability of the emulsion over time, showed significant improvement in the conjugates. CB-EGCG exhibited the highest ESI at 96.23 (min), surpassing CB-TA (87.24 min) and CB at (69.05 min) (*p* < 0.05). The enhanced ESI of CB-EGCG suggests that EGCG strengthens the interfacial protein layer, forming more cohesive and resilient droplet binding, potentially due to strong hydrophobic and selective interactions that stabilize the protein structure and induce the reorganization of the protein network at the interface [29]. Studies have shown that protein–polyphenol conjugates with improved interfacial properties can effectively absorb onto oil droplet surfaces, stabilizing against coalescence through steric hindrance and electrostatic repulsion [30].

#### 2.2.3. Color Characteristic

The color characteristics of chicken muscle protein–polyphenol conjugates are shown in Table 1. The CB control had the highest CIE L* value (lightness), followed by CB-TA and CB-EGCG conjugates. The conjugation with EGCG led to a darker appearance, which was in line with previous research findings that polyphenol conjugation, particularly with catechins such as EGCG, could cause a darker color in protein powders. This could be attributed to the formation of polyphenol–protein networks, decreasing light reflection [31]. Moreover, the decreased lightness in CB-EGCG also corresponded with higher CIE b* (yellowness) and chroma (color saturation), indicating that the color intensity became stronger. Similar observations were noted by Li et al. [32], who found that conjugation with plant polyphenols increased the yellowness of protein powder, likely due to Maillard-like reactions affecting the colorimetric properties. From an industrial perspective, powder with lighter and more neutral colors is generally preferred for food processing, as it can be incorporated into various processed food products, minimizing the adverse impacts on their own color characteristics. Regarding the hue angle, CB-EGCG showed a higher value than the CB control and CB-TA, indicating a slight shift toward yellow–red regions. The whiteness index of the CB-TA conjugate, related to consumer preference for white protein powder, was similar to that of the CB control, implying minimal color change due to conjugation with tannic acid. Chen et al. [33] found that tannic acid could counteract the darkening effect typically seen in protein–polyphenol conjugates by reducing excessive cross-linking, which may benefit manufacturers who prefer lighter-colored powders.

### 2.3. Functional Properties

#### 2.3.1. Antioxidant Capacity

**Figure 5:**
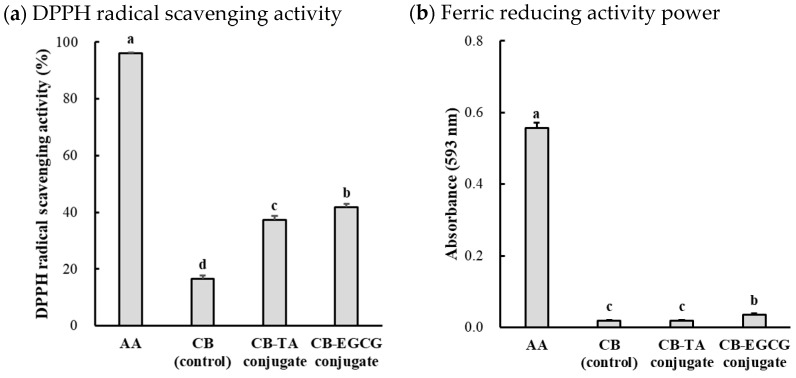
DPPH radical scavenging activity (**a**) and ferric reducing activity power (FRAP, (**b**)) of chicken breast protein–polyphenol conjugates. AA, _L_-ascorbic acid (as positive control); CB, chicken breast powder (as control); CB-TA, chicken muscle protein–tannic acid conjugate; CB-EGCG, chicken muscle protein–EGCG conjugate. a–d Means with the same letters between treatments are not significantly different (*p* > 0.05).

The antioxidant capacity of chicken muscle protein–polyphenol conjugates is shown in Figure 5. The CB-EGCG conjugate exhibited superior antioxidant capacity compared to CB-TA and CB control, as evidenced by its significantly higher DPPH radical scavenging activity and FRAP value. This improvement in CB-EGCG might be attributable to the structural and chemical characteristics of EGCG. EGCG contains multiple hydroxyl groups on its B- and D-rings, which serve as radical scavenging sites [34]. Additionally, EGCG can enhance the radical-neutralizing ability by stabilizing semiquinone radicals through intramolecular hydrogen bonding [35]. Compared to tannic acid, the smaller molecular size and higher affinity of EGCG for reactive protein sites enable the formation of more effective conjugates, maximizing the antioxidant potential of chicken muscle protein–polyphenol conjugates [35]. Similarly, in FRAP analysis (Figure 5b), reflecting the electron-donating ability of samples, the CB control showed negligible reducing power, and the CB-TA conjugate displayed no difference (*p* > 0.05). However, CB-EGCG significantly enhanced FRAP, approximately twice that of the CB control and CB-TA (*p* < 0.05). The higher FRAP of CB-EGCG aligns with the electron-rich rings of EGCG, facilitating electron donation. The high reducing power of CB-EGCG is consistent with the findings of Wang et al. [36], who reported the loss of protons and electrons by activating the phenolic hydroxyl group of EGCG by protein-molecular hydrogen bonds. These conjugates could potentially stabilize the EGCG-induced hydrophobic interactions and the antioxidant-active protein–polyphenol complexes induced by crosslinking. This enhancement could result from structural modifications induced by polyphenols, exposing additional reactive sites on proteins while integrating the intrinsic antioxidant properties of polyphenols [37]. Furthermore, the significant differences between EGCG and tannic acid could indicate the critical role of polyphenol type and structure in determining the antioxidant capacity of resulting conjugates.

#### 2.3.2. In Vitro Digestibility

The in vitro digestibility of chicken muscle protein–polyphenol conjugates was investigated to determine the effects of polyphenol conjugation on protein digestibility (Table 2). The CB control exhibited an in vitro digestibility of 56.77%, and similar in vitro digestibility was also observed for CB-TA (56.27%) and CB-EGCG (57.37%) (*p* > 0.05). This finding suggests that polyphenol conjugation, whether with tannic acid or EGCG, does not substantially affect the overall digestibility of chicken muscle proteins under simulated gastrointestinal conditions. Wen et al. reported that the co-consumption of chicken brisket protein and polyphenols could potentially limit digestibility, with a reduced digestibility of up to 40% observed when the polyphenol concentration ranged from 0–20 mg/mL [38]. However, our findings agreed with previous studies that observed that conjugation with polyphenols can improve protein digestibility despite their co-consumption [39,40]. Similar to our findings, Pomsang et al. recently reported that the conjugation of chicken breast protein with konjac glucomannan hydrolysates increased in vitro bioaccessibility from 58.69% to 66.65% [41]. From a practical perspective, using chicken breast protein–polyphenol conjugates as a protein supplement material could be a convenient way to consume two physiologically beneficial nutrients without compromising bioavailability.

## 3. Materials and Methods

### 3.1. Preparation of Chicken Breast Protein-Polyphenol Conjugates

Chicken breast powder (moisture content of below 7 g/100 g), commercially microwave-vacuum dried, was purchased from a domestic food company (Seobifood Co. Ltd., Namyangju-si, Republic of Korea). The powder (20 mg/mL) was blended with a 10 mM phosphate buffer (pH 7.0), homogenized, and kept at 4 °C for 24 h. The pH of the protein solution was adjusted to 9.0 using a few drops of a 2 M sodium hydroxide solution. To produce protein–polyphenol conjugates, two polyphenol compounds, namely, tannic acid (403040, ACS reagent, Sigma-Aldrich, St. Louis, MI, USA) and epigallocatechin 3-gallate (EGCG, E4143, Sigma-Aldrich, St. Louis, MI, USA), were added at a concentration of 0.1 mg/mL and stirred at room temperature for 24 h. The concentration was selected based on previous observations to maximize conjugation efficiency [42]. During this conjugation process, the sample was exposed to natural air. Subsequently, the conjugate sample was frozen at −70 °C and lyophilized (80 × 10*^−^*^3^ Torr pressure, PVTFD10R, Ilshin Lab, Dongducheon-si, Republic of Korea). The powdered freeze-dried samples were vacuum-packaged in plastic bags and stored at −20 °C until further analysis.

### 3.2. Confirmation and Characterization of Chicken Breast Protein–Polyphenol Conjugates

#### 3.2.1. Determination of Sulfhydryl Groups

The sulfhydryl content of the protein–polyphenol conjugates was measured in duplicate following the method of Fu et al. [43]. Ellman’s reagent was prepared by dissolving 0.2 g of 5,5′-dithiobis-(2-nitrobenzoic acid) in 50 mL of Tris-glycine buffer (Tris 0.086 mol/L, glycine 0.09 mol/L, EDTA 4 mmol/L, and pH 8.0). One milliliter of the sample (1 mg/mL) was mixed with 4 mL of the Tris-glycine buffer and 125 μL of Ellman’s reagent. Following a 1 h reaction at room temperature, the absorbance of the mixture (A_412nm_) was read at 412 nm using a UV-visible spectrophotometer (Biochrom, Libra s22, Cambridge, UK). Sulfhydryl content was calculated using the following equation: Sulfhydryl content (μmol/g) = (73.53 × A_412nm_)/protein concentration (mg/mL)

#### 3.2.2. Turbidity Measurement

The freeze-dried protein–polyphenol conjugate samples were dissolved in deionized distilled water (DDW) (5 mg/mL), and the absorbance was read at 600 nm using a UV-visible spectrophotometer [42].

#### 3.2.3. Zeta Potential

A nanoparticle analyzer (Zetasizer Nano ZS, Malvern, UK) was used to measure the zeta potential of the protein–polyphenol conjugate samples at 25 °C. Prior to measurements, the sample solutions (1 mg/mL) were diluted to deionized distilled water (DDW) [44].

#### 3.2.4. Sodium Dodecyl Sulfate–Polyacrylamide Gel Electrophoresis (SDS-PAGE)

Protein electrophoresis was performed following the method of Laemmli with minor modifications [45]. Protein concentrations of all treatments were diluted to 40 mg/mL. Samples were mixed with the sample buffer (312.5 mM Tris-HCl (pH 6.8), 50% glycerol, 5% SDS, 5% β-mercaptoethanol, and 0.05% bromophenol blue) at a ratio of 4:1 and heat-denatured in a water bath preheated to 100 °C for 5 min. A total of 20 µL of the sample was loaded onto an 8% polyacrylamide gel (5% stacking gel, 8% separating gel), and electrophoresis was conducted using a Mini-Protean Tetra Cell system (Bio-Rad, Hercules, CA, USA). The loaded protein was passed through the stacking gel at 80 V for approximately 15 min and then through the separating gel at 120 V. After electrophoresis, the gel was stained with a staining solution (0.25% (*w*/*v*) Coomassie Blue R-250, 50% (*v*/*v*) methanol, 40% (*v*/*v*) distilled water, and 10% (*v*/*v*) acetic acid) and destained using a destaining solution (50% (*v*/*v*) methanol, 40% (*v*/*v*) distilled water, and 10% (*v*/*v*) acetic acid). A molecular weight marker (AccuLadder™ 3-color Prestained Protein size marker, Bioneer, Daejeon, Republic of Korea) was used to confirm molecular weights.

#### 3.2.5. Fourier Transform Infrared Spectroscopy (FTIR)

Fourier-transform infrared (FTIR) spectra of the conjugated and freeze-dried samples were recorded using a Spectrum Two FT-IR spectrometer (L160000A, PerkinElmer, Shelton, CT, USA) following the method proposed by He et al. [46].

### 3.3. Color Characteristics of Chicken Breast Protein-Polyphenol Conjugates

The instrumental color of the conjugate samples was measured using a Chroma Meter CR-400 (Konica Minolta, Tokyo, Japan). The colorimeter was calibrated with the manufacturer’s standard calibration plate (CIE L*: 93.01, CIE a*: −0.25, CIE b*: +3.50). Samples were placed in Petri dishes with a thickness of over 5 mm, and lightness (CIE L*), redness (CIE a*), and yellowness (CIE b*) values were measured at six random locations. The chroma, hue angle, and whiteness index were calculated as follows [47,48]:

Hue angle=arctangent (b*/a*)

$$Chroma=\sqrt{{a*}^{2}+{b*}^{2}}$$

$$Whitenss\;index=100-\sqrt{{\left(100-L*\right)}^{2}\;{+a*}^{2\;}+{b*}^{2}}$$

### 3.4. Technological Properties of Chicken Breast Protein–Polyphenol Conjugates

#### 3.4.1. Water-Holding Capacity (WHC) and Oil Absorption Capacity (OAC)

The WHC and OAC of the conjugate samples were measured in duplicate following the method of Acosta-Domínguez et al. [49]. One gram of the sample powder was placed in a 15 mL plastic tube and mixed with 10 mL of either DDW or commercial soybean oil (Beksul Co., Seoul, Republic of Korea). The mixture was then centrifuged at 3000× *g* for 10 min at 4 °C. The water or oil supernatant was carefully discarded, and the weight of the residue was measured. The WHC and OHC were expressed as grams of the absorbed water or oil per gram (g/g).

#### 3.4.2. Emulsifying Properties

The emulsifying properties of the conjugate samples were measured following Han et al. [42]. Thirty milliliters of sample solution (5 mg/mL in DDW) was mixed with 10 mL of soybean oil and emulsified for 3 min at 12,000 rpm using a homogenizer (HG-15A, Daihan Sci., Wonju, Republic of Korea). An aliquot of the emulsion (50 μL) was mixed with 10 mL of the 0.1% (*w*/*v*) SDS solution, and absorbance was measured at 500 nm. The emulsifying activity index (EAI) and emulsion stability index (ESI) were calculated using the following equations, with C representing the protein concentration (g/mL), φ representing the oil volume fraction, and A_0min_ and A_30min_ being absorbances at 0 and 30 min, respectively:

EAI (m^2^/g) = 2 × 2.303 × A_0min_ × 200/10,000 C × φ

ESI (min) = A_0min_/(A_0min_ − A_30min_) × 30

### 3.5. Antioxidant Properties of Chicken Breast Protein–Polyphenol Conjugates

#### 3.5.1. 1,1-Diphenyl-2-Picrylhydrazyl (DPPH) Radical Scavenging Activity

The DPPH radical scavenging activity of the protein–polyphenol conjugates was measured in triplicate using the modified method by Chen et al. [50]. One hundred microliters of the 0.1 mM DPPH solution in 99.9% (*v*/*v*) ethanol were mixed with an equal volume of sample solution (1 mg/mL in DDW) or DDW (as control). After the reaction at 25 °C for 30 min, the absorbance of the reactant (A_sample_) was measured at 515 nm using a microplate reader (Infinite M200 PRO, Tecan, Grödig, Austria). _L_-Ascorbic acid (0.5 mg/mL in DDW, PHR1008, Sigma-Aldrich, St. Louis, MI, USA) was used as a positive control. The DPPH radical scavenging activity was calculated using the following equation:

DPPH radical scavenging activity (%) = [1 − (A_sample_ − A_blank_)/A_control_] × 100

#### 3.5.2. Ferric-Reducing Antioxidant Power (FRAP)

The ferric-reducing antioxidant power (FRAP) of the conjugates was assessed in triplicate using the method of Othman et al. [51]. The FRAP reagent was prepared by mixing 300 mM acetate buffer (pH 3.6), 10 mM TPTZ, and 20 mM FeCl_3_·6H_2_O at 37 °C in a 10:1:1 ratio. One hundred microliters of the sample solution (5 mg/mL) were mixed with 3 mL of the FRAP reagent and 300 μL of DDW, and the mixture was kept at 37 °C for 4 min. The absorbance of the reactant was read at 593 nm. _L_-Ascorbic acid (5 mg/mL) was used as a positive control.

### 3.6. In Vitro Digestibility Assessment

In vitro digestibility was assessed in triplicate following the modified method of Bornhorst et al. [52]. For oral digestion, 1 g of the sample powder was mixed with 1 mL of amylase (TB0920, Pickering Laboratories, Mountain View, CA, USA) containing 1.5 mM CaCl_2_. The pH of the sample was adjusted to 7 using 0.1 N NaOH, and the mixture was shaken in a 37 °C water bath for 2 min. For gastric digestion, the oral digest was mixed with simulated gastric fluid (0.03 M NaCl, pH 1.2, 3.2 mg/mL pepsin, 0.15 mM CaCl_2_) at a 1:1 volume ratio, adjusted to pH 3.0, and incubated in a 37 °C water bath for 2 h. For intestinal digestion, the gastric digest was mixed with simulated intestinal fluid (0.05 M KH_2_PO_4_, pH 7.5, 10 mg/mL pancreatin, 0.6 mM CaCl_2_) at a 1:1 ratio, adjusted to pH 7.0, and incubated at 37 °C for 2 h. The final reactant was centrifuged at 3000× *g* for 10 min, and the residue was collected and dried at 65 °C for 12 h to obtain undigested fractions. The in vitro digestibility was calculated based on the weight of the undigested fraction using the following equation.

In vitro digestibility (%) = (100 − (undigested fraction weight (g) × 100)/sample weight (g))

### 3.7. Statistical Analysis

This study was designed as a completely randomized design with a total of three independent replicates. All data are expressed as the mean ± standard deviation. Statistical analyses were performed using SPSS Statistics (Ver. 18.0, IBM, Armonk, NY, USA) with one-way analysis of variance (ANOVA), in which the treatment was fixed as the main effect. Duncan’s multiple range test was used to determine the significance between the means (*p* < 0.05).

## 4. Conclusions

This study demonstrated that polyphenol conjugation enhances the technological properties (e.g., water-holding capacity and emulsion stability) and antioxidant capacity of chicken breast proteins. The type of polyphenol significantly influences these properties by altering the molecular weight and surface characteristics of the protein–polyphenol conjugates. EGCG conjugates exhibited superior radical scavenging activity, ferric-reducing power, and emulsion stability, while tannic acid conjugates improved the water-holding capacity and solubility, making them suitable for protein powders. Moreover, comparable in vitro digestibility was observed, even with the co-digestion of proteins and polyphenols. Our findings indicate the potential of chicken breast protein–polyphenol conjugates as innovative ingredients for high-quality protein supplements. Further research should explore specific protein–polyphenol complexes and their impact on physiological and technological properties. In particular, with the current rapid growth in the market for protein supplements and fortified foods, the introduction of a novel protein ingredient based on the findings of this study could serve as a new strategy for securing market share.

## Figures and Tables

**Table 1:** Color characteristics of chicken muscle protein–polyphenol conjugates.

Trait	CB	CB-TA	CB-EGCG	Significance of *p* Value
CIE L* (lightness)	85.80 ± 0.23 a	84.50 ± 0.08 b	81.55 ± 0.07 c	<0.001
CIE a* (redness)	2.67 ± 0.02 a	2.25 ± 0.02 c	2.40 ± 0.01 b	<0.001
CIE b* (yellowness)	22.30 ± 0.08 b	21.22 ± 0.01 c	25.03 ± 0.02 a	<0.001
Chroma	23.45 ± 0.08 b	21.34 ± 0.01 c	25.14 ± 0.02 a	<0.001
Hue angle	83.47 ± 0.06 b	83.95 ± 0.05 b	84.52 ± 0.03 a	<0.001
Whiteness index	72.59 ± 0.18 a	73.63 ± 0.04 a	68.81 ± 0.03 b	<0.001

CB, chicken breast powder (as control); CB-TA, chicken muscle protein–tannic acid conjugate; CB-EGCG, chicken muscle protein–EGCG conjugate. a–c Means sharing the same letters in a row are not significantly different (*p* > 0.05).

**Table 2:** In vitro digestibility of chicken muscle protein–polyphenol conjugates.

CB	CB-TA	CB-EGCG	Significance of *p* Value
56.77 ± 0.06	56.27 ± 2.37	57.37 ± 2.07	0.771

CB, chicken breast powder (as control); CB-TA, chicken muscle protein–tannic acid conjugate; CB-EGCG, chicken muscle protein–EGCG conjugate.

## Data Availability

The original contributions presented in the study are included in the article; further inquiries can be directed to the corresponding author.
